# Exonal Elements and Factors Involved in the Depolarization-Induced Alternative Splicing of Neurexin 2

**DOI:** 10.1007/s12031-012-9919-x

**Published:** 2012-11-21

**Authors:** G. Rozic, Z. Lupowitz, N. Zisapel

**Affiliations:** Department of Neurobiology, The George S Wise Faculty of Life Sciences, Tel Aviv University, Tel Aviv, 69978 Israel

**Keywords:** Neurexin, Splicing, Depolarization, hnRNP K, hnRNP L

## Abstract

The neurexin genes (NRXN1, NRXN2, and NRXN3) encode polymorphic presynaptic proteins that are implicated in synaptic plasticity and memory processing. In rat brain neurons grown in culture, depolarization induces reversible, calcium-dependent, repression of NRXN2α exon 11 (E11) splicing. Using Neuro2a cells as a model, we explored E11 *cis* elements and *trans*-acting factors involved in alternative splicing of NRXN2α E11 pre-mRNA under basal and depolarization conditions. E11 mutation studies revealed two motifs, CTGCCTG (enhancer) and GCACCCA (suppressor) regulating NRXN2α E11 alternative splicing. Subsequent E11 RNA affinity pull-down experiments demonstrated heterogeneous nuclear ribonucleoprotein (hnRNP) K and hnRNP L binding to this exon. Under depolarization, the amount of E11-bound hnRNP L (but not of hnRNP K) increased, in parallel to NRXN2α E11 splicing repression. Depletion of hnRNP K or hnRNP L in the Neuro2a cells by specific siRNAs enhanced NRXN2α E11 splicing and ablated the depolarization-induced repression of this exon. In addition, depolarization suppressed whereas hnRNP K depletion enhanced NRXN2α expression. These results indicate a role for hnRNP K in regulation of NRXN2α expression and of hnRNP L in the activity-dependent alternative splicing of neurexins which may potentially govern *trans*-synaptic signaling required for memory processing.

## Introduction

Neurexins are a family of synaptic proteins that are implicated in cognitive functioning. In mammals, neurexins are encoded by three independent genes, NRXN1, NRXN2, and NRXN3, each transcribed from two promoters to yield a long form (α) or a short form (β) and through differential use of five alternative splice sites yield over 1,000 different isoforms (Ushkaryov et al. 1992, 1994; Ullrich et al. 1995; Tabuchi and Sudhof 2002). NRXN2α is linked to risk of autism spectrum disorder (Gauthier et al. 2011) and response to external insults and ischemia (Gorecki et al. 1994; Sun et al. 2000; Kattenstroth et al. 2004).

We have previously found in day-old rat brain neurons grown in culture, depolarization (55 mM KCl) induced repression of the alternative splicing of exon 11 (E11) in NRXN2α and NRXN3α but not in NRXN1α transcripts (Rozic-Kotliroff and Zisapel 2007; Rozic et al. 2011). Further in vivo studies demonstrated that changes in transcription and splicing of NRXNs were dynamic and potentially involved in synaptic remodeling occurring at an intermediate (hours) time scale in the course of memory formation (Rozic-Kotliroff and Zisapel 2007; Rozic et al. 2011) and in coupling the circadian clock to diurnal rhythms in excitatory/inhibitory synaptic balance (Shapiro-Reznik et al. 2012). Delineating the molecular control of E11 NRXN2α splicing may help elucidate the link between neuronal activity and choice of alternative exons. Because of the role of neurexins in synaptic plasticity and cognition, such knowledge may help define the molecular control of dynamic, activity-dependent changes in synaptic strength that occur in the adult brain, where they are thought to underlie learning and memory formation (Murase and Schuman 1999).

Depolarization-induced changes in alternative splicing have now been observed in a number of genes (review in (Xie 2008)) but the molecular control of such changes is not well understood (An and Grabowski 2007; Lee et al. 2007; Yu et al. 2009).

Alternative splicing patterns are mediated by specific proteins that bind to regulatory enhancer and silencer elements in the pre-mRNAs (Cartegni et al. 2002; Fairbrother et al. 2002; Black 2003; Zhang and Chasin 2004) thereby acting as splicing activators and repressors, respectively (Black 2003). The aim of the present study was to identify exonal *cis* elements and *trans*-acting factors mediating the NRXN2α E11 splicing repression. The studies were performed in Neuro2A neuroblastoma (Neuro2A) cells to provide homogenous neuronal-derived cell population and facilitate plasmid transfection experiments. Furthermore, these cells demonstrate depolarization-induced NRXN2α E11 splicing repression and E11 binding proteins demonstrated in primary brain neurons in culture as is shown below.

## Experimental Procedures

### Cell Culture and Treatment

Neuro2A cells seeded in six-well culture plates (1.5–4 × 10^5^ cells per well) were maintained in DMEM supplemented with 10 % fetal calf serum, 100 units/ml penicillin, 100 μg/ml streptomycin, and 2 mM glutamine (Biological Industries) at 37 °C and 5 % CO_2_ for 24 h. The cells were incubated with 50 mM (“high K”) or 5 mM (“low K”) KCl in growth medium for 4 h (found in optimization studies to ensure a fully developed response). SCN2.2 cells were grown in culture as described previously (Shapiro-Reznik et al. 2012).

### Minigene Construction

Oligonucleotide primers were designed to amplify exons 10, 11, and 12 of the NRXN2α gene and their flanking introns from rat genomic DNA (obtained using the Genomic DNA Purification kit from Sigma). Each primer contained an additional extension encoding a restriction enzyme sequence. The PCR products were digested with Hind III/Kpn I and inserted between the Hind III/KpnI (exon 10 and flanking intron) and Kpn I/BamH I (exons 11 and 12 and flanking introns) sites in the pEGFP-C1 vector (Clontech). The spliced cDNA products derived from the expressed minigene were detected by PCR using pEGFP-C1 forward primer and exon 12 reverse primers as follows:

**Table:** 

Minigene construction:	
Intron 9 Hind III forward	5′-CCTAGAAGCTTCCTTTGCCCCTCTTCTCCACTCC
Intron 10-11 Kpn I reverse	5′-CCATCGGTACCTTGGTGAACTGGTCTCCTCCC
Intron 10-11 Kpn I forward	5′-GCCATGGTACCGCTGAGATTGCCAAGGTGCCCG
Intron 12-13 BamH I reverse	5′-GGTAAGGATCCGCTGAAATGGTCCCTAACGTCTCTGG
Minigene detection:	
pEGFP forward	5′-CGGCATGGACGAGCTGTAC
NRXN2 E12 reverse	5′- CTTGTGCCCCGCAAAGAG
M1 forward	5′-CATGGCCCCCCCAG**T**CTGCCTGCGCGTCG

### Minigene Mutations

Site-directed mutagenesis was performed in the minigene using the i-pfu DNA polymerase (Talron Biotech L.T.D.). Amplification reactions (PTC-200 thermal cycler) started with denaturation phase (3 min at 95 °C) followed by 16 cycles of incubations (30 s at 95 °C, 1 min at 55 °C, and 16 min at 72 °C) using the following mutagenic primers.

**Table:** 

M1 reverse	5′-CGACGCGCAGGCAG**A**CTGGGGGGGCCATG
M2 forward	5′-GCCCCCCCAGACTGC**T**TGCGCGTCGGCTGC
M2 reverse	5′-GCAGCCGACGCGCA**A**GCAGTCTGGGGGGGC
M3 forward	5′-CAGACTGCCTGCGCG**AG**GGCTGCGCACCCAG
M3 reverse	5′-CTGGGTGCGCAGCCC**TC**GCGCAGGCAGTCTG
M4 forward	5′-GCCTGCGCGTCGGCT**AA**GCACCCAGTAAGTG
M4 reverse	5′-CACTTACTGGGTGC**TT**AGCCGACGCGCAGGC

### Minigene Transfection and RNA Isolation

The cells were transfected with reporter plasmids using TransIT®-LT1 transfection reagent according to the manufacturer’s instructions (Mirus). Twenty-four hours after transfection, total RNA was extracted using EZ-RNA Total RNA Isolation Kit.

### siRNA Knockdown, RNA Isolation, and RT-PCR Analysis

Multiple siRNA duplexes specific for mouse heterogeneous nuclear ribonucleoprotein (hnRNP) K, hnRNP L, and scrambled negative control siRNAs as mock transfections (IDT technologies) were transfected into the cells using X-tremeGENE siRNA transfection reagent according to the manufacturer’s instructions. Seventy-two hours after transfection, the cells were incubated with 5 or 50 mM KCl for 4 h and total RNA and proteins (nuclear and cytosolic fractions) were isolated. The following siRNA duplexes were used:

**Table:** 

hnRNP K	MMC. RNAI.025279.8.1 (K1)	5′-GCCUAUUGGUGGAUCCAUUUAAUTC
	MMC. RNAI.025279.8.6 (K2)	5′-GGGUUGUAGAAUGCAUCAAGAUCAT
	MMC. RNAI.025279.8.10 (K3)	5′-AGGAGGCAAGAAUAUUAAGGCUCTC
hnRNP L	MMC. RNAI.N177301.8.1 (L3)	5′-GAACGAUCAAAGAUACUUGGGACTA
	MMC. RNAI.N177301.8.3 (L2)	5′-AGGCUUAACCACAACAGAAAUGCTG
	MMC.RNAI.N177301.8.5 (L1)	5′- GGCCCUGUCCAGAGAGAAUUGUCAUTT
Negative control	Scrambled negative control (scr1)	5′-CUUCCUCUCUUUCUCUCCCUUGUG
	NC1 negative control (scr2)	5′-CGUUAAUCGCGUAUAAUACGCGUAT

### PCR Amplification

Total RNA (1 μg) was primed by oligo dT and reverse-transcribed by Verso cDNA kit (Zotal, Israel) according to the manufacturer’s instructions. cDNA sample aliquots were added to reaction mixtures containing 1.5 mM MgCl_2_, 200 μM dNTP, 500 nM of each primer, and 1 U *Taq* DNA polymerase. Amplification reactions (PTC-200 thermal cycler) started with denaturation phase (3 min at 94 °C) followed by repeated cycles of incubations (30 s at 94 °C, 30 s at 62 °C, and 30 s at 72 °C). PCR products were subjected to electrophoresis on 2.5 % agarose gel and stained with ethidium bromide (10 μg/ml). Gels were photographed on top of a 280-nm UV light box. The following primers were used:

**Table:** 

GFP forward	5′-CGGCATGGACGAGCTGTAC
E12 NRXN2 reverse	5′-CTTGTGCCCCGCAAAGAG
Actin forward	5′-GCCCTAGACTTCGAGCAAGAGA
Actin reverse	5′-CCAGGATAGAGCCACCAATC

### Real-Time PCR analysis

When depolarization of Neuro2A cells was performed, 48 h after transfection, the cells were exposed to KCl 5 or 50 mM for 4 h and total RNA and proteins were isolated. Total RNA (1 μg) was primed by oligo dT and reverse-transcribed by Verso cDNA kit (Zotal) according to the manufacturer’s instructions. Gene expression values were calculated based on the comparative threshold cycle (Ct) method using the following primers:

**Table:** 

E11 NRXN2 endogenous expression:	
E11 NRXN2 forward	5′-CTGCCATTGCCTCCTGAGG
E11 NRXN2 reverse	5-CAGCCGACGCGCAGG
NRXN2 forward	5′-TCCAGGGACCCAGGCAAC
NRXN2 reverse	5′-GCTCAGGCCACCGATATAC
E11 NRXN2 minigene expression:	
E11 forward	5′-CTGCTGGAGTTCGTGACCGC
E11 reverse	5′-CAGCCGACGCGCAGG
GFP forward	5′-GAGCAAGGGCGAGGAGC
GFP reverse	5′-CCTGGACGTAGCCTTCGG
Other genes	
GAPDH forward	5′-GACAACTTTGGCATCGTGGA
GAPDH reverse	5′-ATGCAGGGATGATGTTCTGG
hnRNP K forward	5′-AGACCGAACAGCCAGAAGAA
hnRNP K reverse	5′-TCCAGCATTCTTGCTCTGAA
hnRNP L forward	5′-GGAAATGGCTGATGGCTATG
hnRNP L reverse	5′-ACCGATTGTTCCTTGACTCG
SRp40 forward	5′-CCAGATCAGTTGACAGTGG
SRp40 reverse	5′-GGTGGTCCACATCTACAAA

The Ct data for E11 NRXN2 isoform, total NRXN2 mRNA, and the reference gene GAPDH mRNA in each sample were used to create ΔCt values for total NRXN2 in sample (Ct_total NRXN2_ − Ct_GAPDH_) and E11 NRXN2 including transcripts (Ct_E11 NRXN2 including transcripts_ − Ct_total NRXN2 or GAPDH_). Thereafter, ∆∆Ct values were calculated by subtracting the ∆Ct value of the untreated control sample from the ∆Ct value of treated sample and expressing Rq using the formula $\mathrm{Rq}={2^{{-\varDelta \varDelta \mathrm{Ct}}}}$.

### Biotin-RNA Pull-down Assay

E11 NRXN2α RNA containing biotin at 5′-position was used in the pull-down assay as follows:

**Table:** 

E11 NRXN2	5Biosg/rCrCrCrCrArGrArCrUrGrCrCrUrGrCrGrCrGrUrCrGrGrCrUrGrCrGrCrArCrCrCrArGrUrArArGrU

Nuclear proteins were extracted using CelLyticNuCLEAR Extraction Kit (Sigma) according to the manufacturer’s instructions. The RNA (15 μg) was incubated with 500 μg of nuclear proteins for 30 min at 30 °C in a binding buffer containing 10 mM HEPES pH 7.6 NaOH, 3 mM MgCl2, 5 mM EDTA, 40 mM KCl, 2 mM DTT, 5 % glycerol, 0.5 % NP40, RNAse inhibitor, and 400 μg/ml tRNA. Following binding, the reaction mixtures were placed on ice and UV irradiated at 254 nm at a distance of 10 cm for 30 min. Then 30 μl of streptavidin–agarose beads (Sigma) were added to the reaction and incubated at 4 °C overnight. Prior to this step, the original streptavidin–agarose bead preparation was pre-adsorbed with 1 mg/ml of bovine serum albumin and 400 μg/ml/ml tRNA, for 30 min at 4 °C. The beads were washed three times and resuspended in 300 μl of the binding buffer. The protein–RNA–streptavidin–agarose complex was washed five times with the binding buffer, eluted by boiling at 95 °C for 5 min in 30 μl of SDS sample buffer (2 % SDS, 80 mM Tris-HCl, 5 % β-mercaptoethanol,15 % glycerol, 0.05 % bromophenol blue, pH 6.8), resolved by SDS-PAGE (10 % acrylamide gel), and stained with Coomassie Brilliant blueR-250. The specific protein bands were excised and identified by mass spectrometry (Smoler Proteomic Center, Technion).

### Immunoblotting

The protein samples (25 μg) were subjected to SDS-PAGE and immunoblotting. The levels of hnRNP K, hnRNP L, green fluorescence protein (GFP), and actin were determined using specific primary antibodies to hnRNP K(ab52600), hnRNP L (4D11), GFP(ab290) (all from Abcam, Cambridge, MA), and anti-β-actin (from MP Biomedicals, Solon, Ohio), followed by the secondary antibodies conjugated to IRDye 800 or IRDye 680 DX (LI-COR Biosciences) diluted 1:10,000 in PBS. Each detected band was quantified using the Odyssey Infrared Imaging System Odyssey and imaging software 3.0 (LI-COR Biosciences) and normalized to the level of actin in the corresponding lanes. The fold increase of a specific protein was obtained from the ratio of the respective bands’ intensities in samples from treated and untreated control cells.

### Immunostaining

Cells (3 × 10^5^) plated onto glass cover slips were fixed with 4 % paraformaldehyde in phosphate-buffered saline; incubated with goat γ globulin (Jackson ImmunoResearch Laboratories, PA, USA), 200 μg/ml, 30 min, followed by the rabbit anti-hnRNP K for 1 h and Alexa flour® 546 Donkey anti-rabbit (Molecular Probes, Invitrogen, Oregon, USA); stained with 10 μg/ml Hoechst 33258 dye (Sigma-Aldrich, MO, USA); and subjected to confocal microscopy imaging.

### Data Analysis

All data presented in the figures are representative of two to three experiments in triplicates. A two-sided *t* test between groups was performed using the Excel package for Windows 2010 (Microsoft). Differences between treatment groups were judged to be statistically significant at *p* < 0.05.

## Results

### Depolarization-Induced NRXN2 E11 Splicing Repression in Neuro2a Cells

**Fig. 1:**
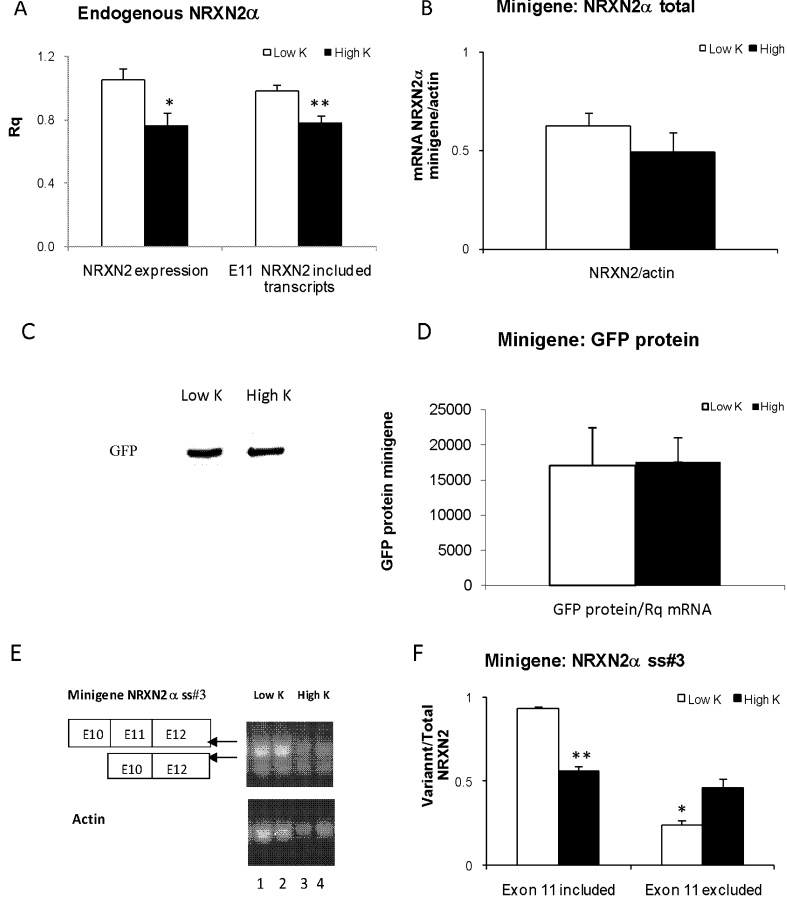
Effects of depolarization on endogenous NRXN2α and on transfected minigene expression and SS#3 splicing in Neuro2a cells. Native cells or cells transfected with the minigene were incubated with 5 mM (low K) or 50 mM (high K) KCl for 4 h. **a** Endogenous NRXN2α total mRNA and E11 including transcripts were assessed by real-time PCR. **b** Minigene mRNA products were quantified using PCR. **c**, **d** Minigene GFP protein relative to RNA content was quantified by immunoblotting using anti-GFP antibody. **e**, **f** Quantification of the minigene transcripts was expressed relative to the sum of the E11 included and excluded RT-PCR products (mean + SE from two experiments (triplicates)). The use of PCR was selected to demonstrate the decrease in E11 included and concomitant increase in E11 excluded variants. Gene expression values were calculated using the comparative threshold cycle method as described in “Experimental Procedures”.*p < 0.05, **p < 0.001

In Neuro2a cells, nearly 90 % of the NRXN2α transcripts included E11. Depolarization of the cells led to a significant decrease in the expression of NRXN2α compared to the actin housekeeping gene and a significant repression ofNRXN2α E11 splicing leading to a decrease in E11 including transcripts (Fig. 1a). A minigene reporter plasmid, comprising exons 10, 11, and 12 of the NRXN2α gene and their flanking introns linked to GFP gene, was constructed to investigate the potential role of NRXN2α E11 sequence elements in splicing regulation of this exon. The effects of depolarization (50 mM KCl, 4 h) on E11 splicing in Neuro2a cells transiently transfected with the minigene were studied 24 h after transfection. Depolarization did not affect significantly NRXN2α minigene transcripts levels (Fig. 1b) and respective GFP- coupled protein products (Fig. 1c, d). Reverse transcription (RT-PCR) of NRXN2α mRNA followed by polymerase chain reaction in Neuro2a cells transiently transfected with the minigene indicated that, as with the innate NRXN2α, under non-stimulated conditions (5 mM KCl) 90 % of the minigene transcripts included the E11 exon (Fig. 1e, lanes 1 and 2). Depolarization resulted in significant repression of NRXN2α minigene E11 splicing (Fig. 1e, lanes 3 and 4; Fig. 1f).

### Role of E11 *cis* Elements in the Splicing Regulation

**Fig. 2:**
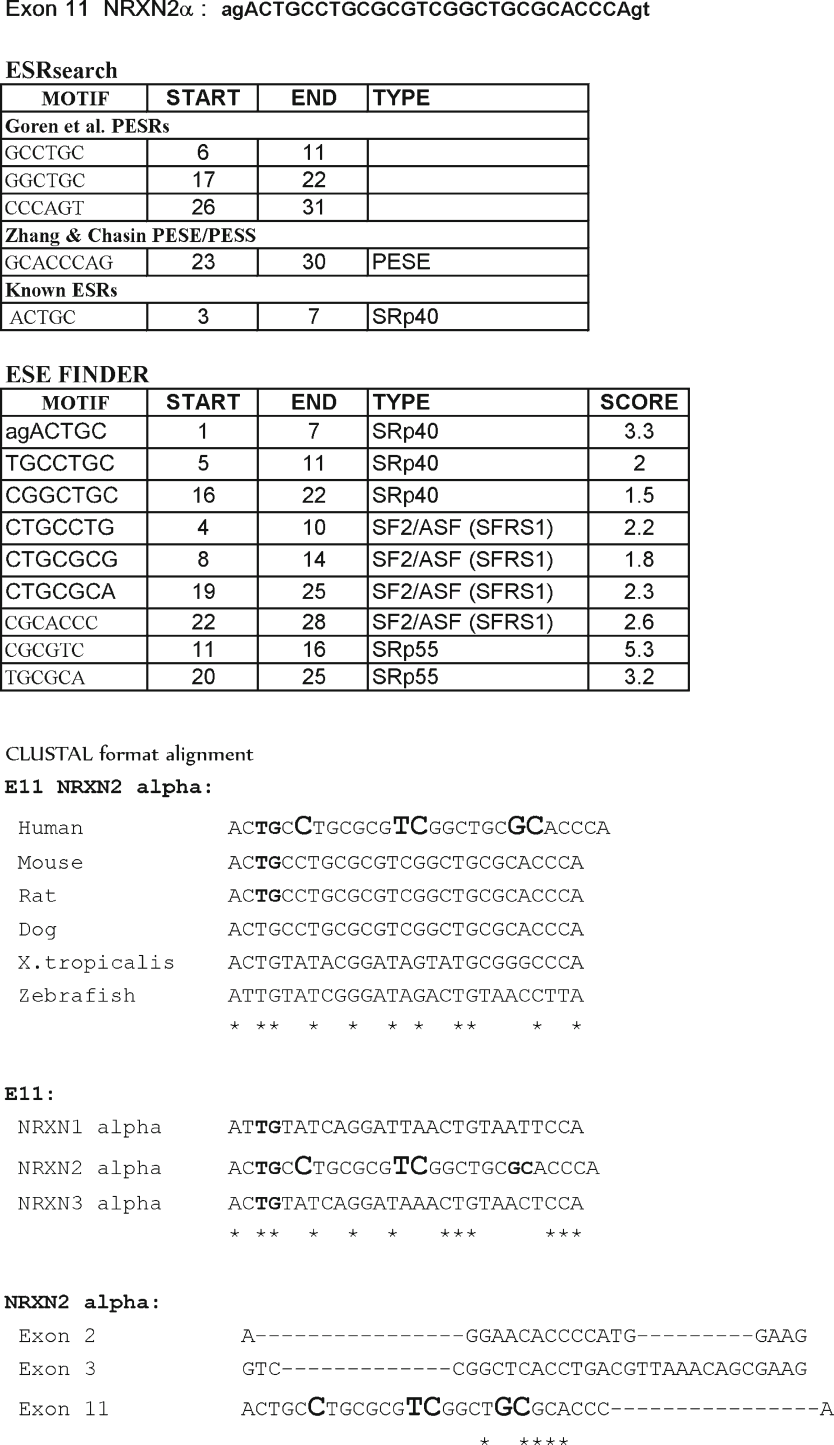
E11 NRXN2αgene alignment with motifs identified by ESE finder, ESRsearch, Web interfaces, and Clustal W multiple sequence alignment

Site-directed mutagenesis was designed, using several strategies, to identify putative *cis* elements involved in splicing regulation of NRXN2α E11. First, we identified high-score putative RNA binding sequences in the exon using the ESE finder (Cartegni et al. 2003; Smith et al. 2006) and ES Research Web interfaces (Fairbrother et al. 2002; Wang et al. 2004; Zhang and Chasin 2004; Goren et al. 2006). This approach highlighted an element (GCACCCAG) located at the 3′ end of E11, as a putative PESE/hnRNP K binding site, another element (ACTGC) located close to the 5′ end of E11, as a putative SRp40 binding motif, CTGCCTG a putative SF2/ASF and SRp40 binding site and CGCGTC a putative SRp55 and SRp40 binding site (Fig. 2).

The second approach considered that although the amino acids sequence encoded by NRXN2α E11 is 100 % conserved among mammals, NRXN2α E11 shares 48 % homology with NRXN3α E11 and 41 % homology with NRXN1α E12 at the nucleotide level, whereas NRXN1α E12 shares 85 % homology with NRXN3α E11 (Fig. 2). Since in cortical neurons the depolarization-induced E11 splicing repression was most pronounced in NRXN2α, less so in NRXN3α and did not occur at all in NRXN1α, we have mutated NRXN2α E11 by replacing nucleotides that are shared with NRXN3α E11 but not with NRXN1 αE12 (Fig. 2). The third approach considered that in addition to E11, the splicing of NRXN2α exons 2 (E2) and 3 (E3) were also repressed by high KCl treatment (Rozic-Kotliroff and Zisapel 2007). We have designed mutations in the homologous domain shared by NRXN2α E2, NRXN2α E3, and NRXN2α E11 (CACC, Fig. 2).

**Fig. 3:**
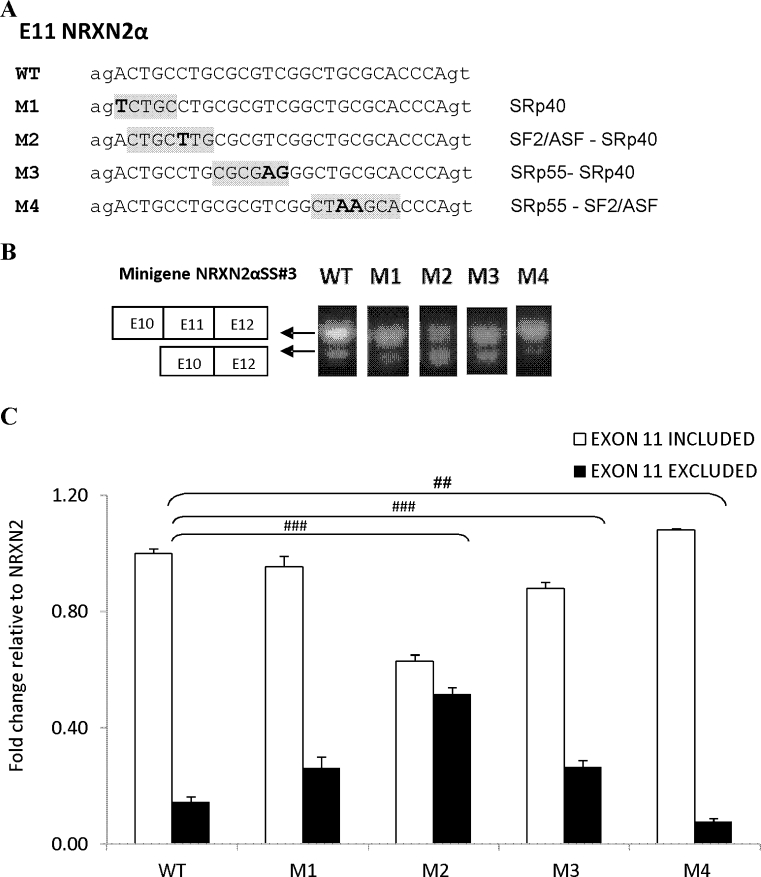
Effects of E11 NRXN2α mutations on splicing patterns. **a** Sequence of the E11 NRXN2α is shown, with engineered mutations. Putative splicing factors reported to interact with these sequences are indicated. The mutated nucleotides are shown in *bold letters* and motifs affected by the mutation are denoted by *gray shades*. **b** Agarose gel electrophoresis of the RT-PCR products after transient transfection of the wild-type (WT, *lane 1*) and mutant splicing reporter plasmids (M1–M4, *lanes 2–5*) in Neuro2a cells. **c** Quantification of the bands using the Image J program. Each transcript is expressed relative to the sum of both RT-PCR products. (mean + SE from at least three independent experiments).*p < 0.05, **p < 0.01, ***p < 0.005

Based on these approaches, four mutant minigenes (M1–M4) were constructed (Fig. 3a). The effects of these mutations on NRXN2α E11 splicing in the Neuro2a cells under basal conditions (low K) are depicted in Fig. 3. In cells transfected with the mutant minigene constructs, expression of E11 including transcripts was unaffected by M1 mutation (targeting a SRp40 binding motif) compared to cells transfected with the wild-type (WT) minigene. However, E11 splicing was markedly reduced (2.6-fold, *p* = 1.65E-08) by the M2 mutation targeting a putative ASF/SF2-SRp40 binding motif and to a lesser extent (1.6-fold, *p* = 0.0013) by the M3 mutation, targeting a putative SRp55-SRp40 binding motif. M4 mutation targeting putative PESE and hnRNP K binding motifs enhanced E11 splicing in the cells (Fig. 3b, c).

**Fig. 4:**
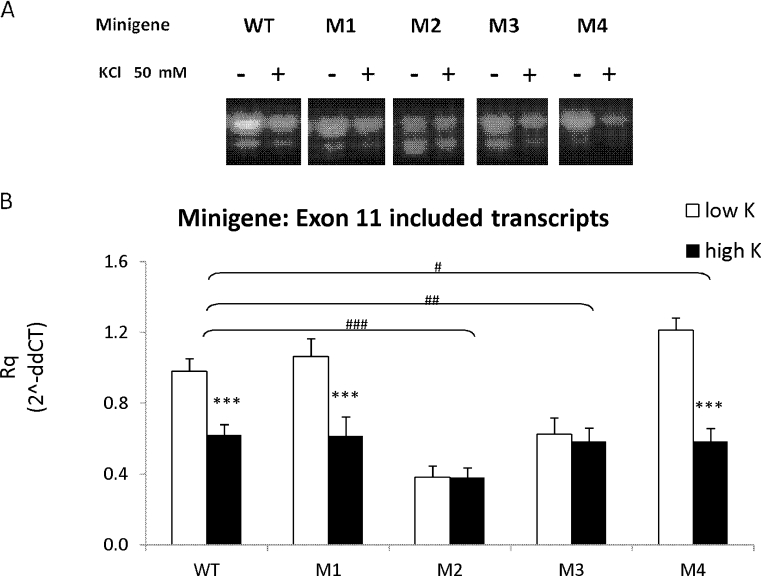
Effects of E11 NRXN2α mutations on the depolarization-induced repression of exon 11 inclusion transcripts. Agarose gel electrophoresis of the RT-PCR products after transient transfection of the wild-type and mutant splicing reporter plasmids M1–M4, in Neuro2a cells and treatment with low and high K for 4 h. **b** The amount of E11 minigene including transcripts was assessed by real-time PCR. (mean + SE from at least two independent experiments in triplicates). Gene expression values were calculated using the comparative threshold cycle method as described in “Experimental Procedures”. *p < 0.05, **p < 0.01, ***p < 0.005

The effects of depolarization on E11 splicing in Neuro2a cells transfected with the WT and mutant minigenes were also studied (Fig. 4). As expected, M2 and M3 mutations that already repressed E11 splicing obviated the depolarization-induced repression of the NRXN2α minigene E11 splicing, whereas M4 mutation that enhanced E11 splicing under basal conditions did not prevent the high KCl-induced NRXN2α E11 splicing repression.

### Identification of the E11 NRXN2α RNA Binding Proteins

**Fig. 5:**
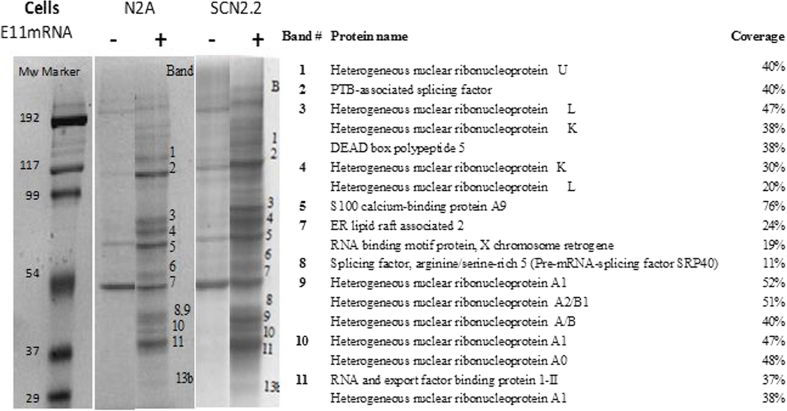
Identification of RNA binding proteins interacting with E11 NRXN2α RNA. **a** Neuro2a cells were incubated without (*lane 1*) and with (*lane 2*) biotin-labeled E11 NRXN2α RNA. **b** The specific protein bands (numbered consecutively) were excised and identified by mass spectrometry. **c** The level of hnRNP L, hnRNP K, and SRp40 mRNA transcripts in Neuro2a cells before and after depolarization was assessed by real-time PCR. (Mean + SE from at least two independent experiments in triplicates). Gene expression values were calculated using the comparative threshold cycle method as described in “Experimental Procedures”

Having found that the exon includes putative splicing motifs, we then sought to identify splicing *trans*-acting factors that interact with NRXN2α E11 motifs. This was pursued using NRXN2α E11 biotinylated RNA as bait for RNA binding proteins extracted from the Neuro2A cells nuclei. To evaluate nonspecific binding, nuclear proteins were incubated with the beads in the absence of biotinylated E11 NRXN2α RNA. As can be seen in Fig. 5, SDS-PAGE analysis of the pre-absorbed proteins eluted from the biotin-labeled beads identified nearly 20 different protein bands. Of these, 11 bands that bound specifically in the presence but not absence of the biotinylated E11 NRXN2α RNA were identified by mass spectrometry (MALDI-TOF) and found to comprise, among others, hnRNP L and hnRNP K forms (bands 3 and 4) and serine/arginine residue SRp40 (band 8) in agreement with the bioinformatic analysis. In immortalized rat SCN2.2 neurons in vitro (Earnest et al. 1999), the E11 mRNA binding proteins in SCN2.2 cells nuclei were essentially the same as those found in Neuro2a cells (Fig. 5). These cells retain most biochemical and functional properties of the circadian clock with internal periodicity of ∼24 h (Womac et al. 2009).

### Effects of Depolarization on E11 NRXN2α RNA Binding Proteins

**Fig. 6:**
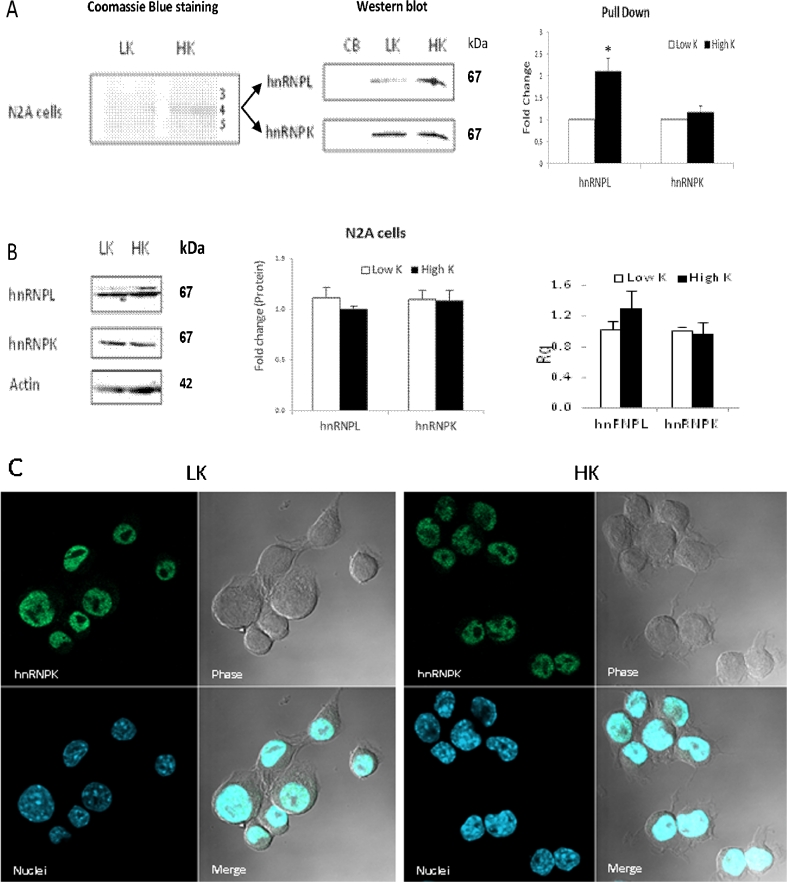
Effect of depolarization on E11 NRXN2α RNA binding proteins. Neuro2a cells treated with 5 mM (low K) and 50 mM KCl (high K) for 4 h. **a** Levels of hnRNP K, hnRNP L, and SRp40 mRNA levels in the cells were quantified by real-time PCR. Gene expression values were calculated using the comparative threshold cycle method as described in “Experimental Procedures” (mean + SE from two experiments in triplicates).*p < 0.05, **p < 0.01, ***p < 0.005. **b** Nuclear extracts were incubated with biotin-labeled E11 NRXN2α RNA and resolved by SDS-PAGE followed by staining with Coomassie Brilliant blueR-250(*left panel*) or immunoblotting using anti-hnRNP L and hnRNP K antibodies (*middle panel*). Immunoblot data were quantified (*right panel*, mean + SE fold change relative to levels at low K; data from two experiments). **c** Levels of hnRNP K and hnRNP L in the nuclear extracts from the cells were quantified by immunoblotting (*left panels*, mean + SE from three experiments). **d** Localization of hnRNP K was assessed by immunostaining

There were no differences in innate mRNA transcript levels of hnRNP L and hnRNP K in Neuro2a cells under high and low KCL conditions (Fig. 5b). Similarly, the total amounts of hnRNP K and hnRNP L in the nucleus were not affected by the high KCl treatment, as indicated by immunoblots of the nuclear extracts from the cells (Fig. 6b) and confirmed by immunostaining (Fig. 6c). However, there was an increase in the amount of hnRNP L that bound to the biotinylated E11 NRXN2α RNA bait in nuclear extracts of depolarized as compared to non-depolarized Neuro2a cells, whereas the amount of hnRNP K that bound to the bait did not change (Fig. 6a). No effect of depolarization on SRp40 mRNA expression in the Neuro2a cells was found (data not shown). Under depolarizing conditions, the amount of E11-bound SRp40 as also most of the other nuclear proteins which interacted with the biotinylated E11 bait decreased (data not shown).

### Effect of hnRNP L Knockdown on NRXN2α E11 Splicing

**Fig. 7:**
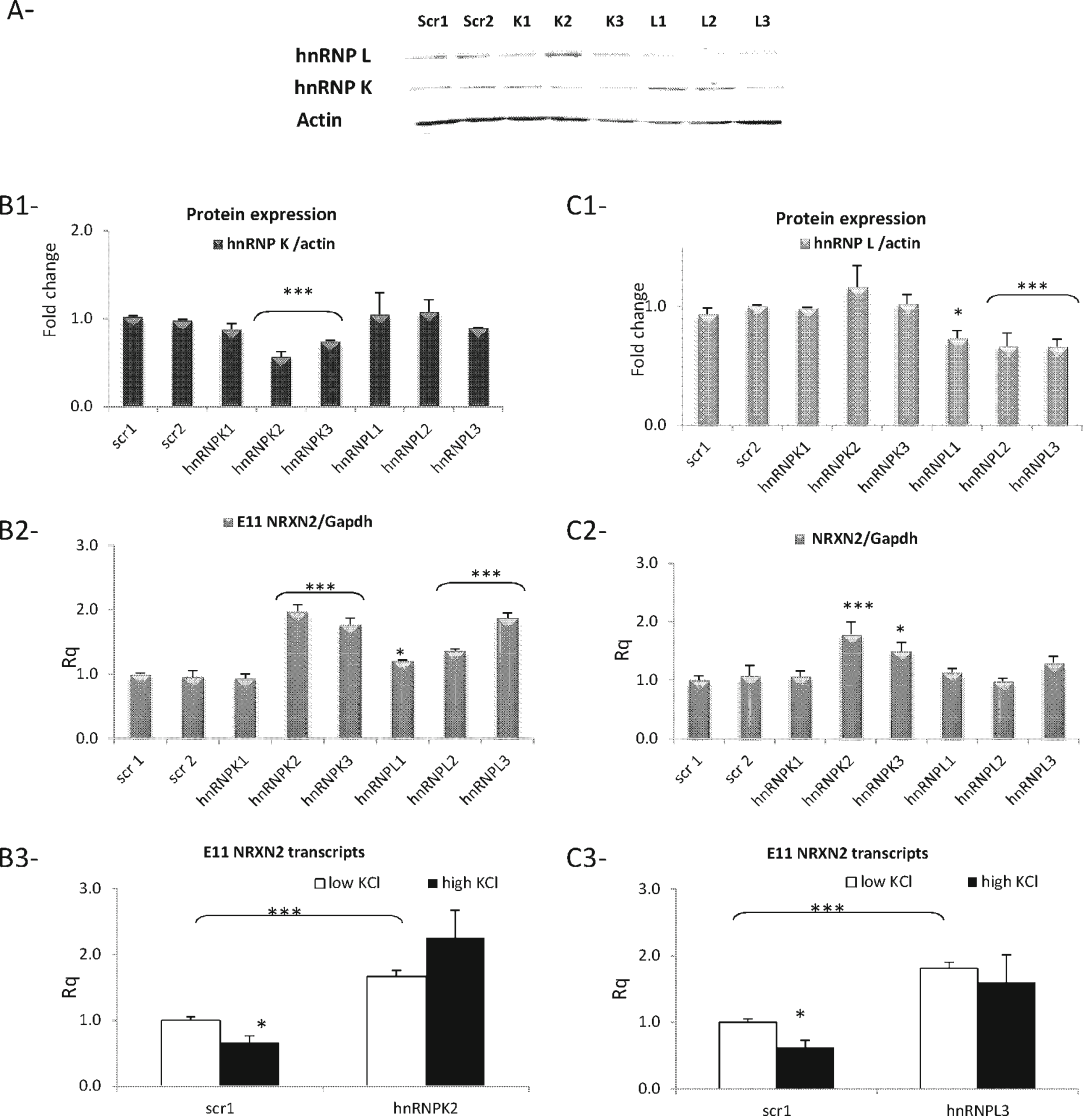
Effect of hnRNP K siRNA knockdown on E11 NRXN2α splicing. Neuro2A cells co-transfected with the minigene and siRNAs directed against scrambled sequences as negative control (scr1, scr2), hnRNP K (K1, K2, K3), or hnRNP L (L1, L2, L3) for 72 h. The proteins were extracted and analyzed by immunoblotting using anti-hnRNP K or hnRNP L antibodies (*A*) and quantified by Odyssey imaging software (*B1*, *C1*). The amount of E11 NRXN2α including transcripts and NRXN2α expression was quantified by real-time PCR (*B2*, *C2*). The cells were incubated with 5 mM (low K) and 50 mM (high K) concentrations for 4 h. The amount of E11 NRXN2α including transcripts was quantified by real-time PCR (*B3*, *C3*). Gene expression values were calculated using the comparative threshold cycle method as described in “Experimental Procedures” (mean + SE from two experiments in triplicates with each siRNA).*p < 0.05, **p < 0.01, ***p < 0.005

The effect of hnRNP L knockdown on E11 splicing in the Neuro2a cells was explored using three different siRNAs (L1, L2, and L3) and scrambled siRNA controls (Fig. 7, A). As can be seen in Fig. 7, hnRNP L protein levels decreased significantly in cells transfected with each of the three siRNAs (72 h after transfection) compared to scrambled siRNAs control. In parallel, the amount of NRXN2α E11 including transcripts increased under basal conditions, and the depolarization-induced splicing repression was abolished in the hnRNP L depleted cells. Total NRXN2α expression was not affected (Fig. 7, B, C).

### Effect of hnRNP K Knockdown on NRXN2α E11 Inclusion in Neuro2A Cells

The effect of hnRNP K knockdown on E11 splicing in the Neuro2A cells was explored using three specific siRNAs (K1, K2, and K3) and scrambled siRNA controls (Fig. 7, A). As can be seen in Fig. 7, B1, hnRNP K protein levels (72 h after transfection) decreased significantly in cells transfected with two (K2 and K3 but not K1) of the three siRNAs compared to scrambled siRNAs controls. In parallel, the amount of NRXN2α E11 including transcripts increased significantly in the hnRNP K depleted cells (K2 and K3 but not K1) and depolarization-induced splicing repression was abolished. Total NRXN2α expression increased as compared in these cells (Fig. 7, B, C).

## Discussion

NRXNα alternative splicing sites are located within laminin neurexin sex hormone-binding protein (LNS) domains, named LNS2, LNS4, and LNS6 corresponding to exons 6, 11, and 20, respectively (Tabuchi and Sudhof 2002). Crystal data of most of the extracellular sequences of neurexins 1α (i.e., neurexin 1α with and without Ca2+, and with and without splice insert at #SS3) have recently been published (Chen et al. 2011; Miller et al. 2011). The structures of the three forms are remarkably similar, in that each of the five LNS domains contain an identically folded core, but individual surface loops give each domain a putatively unique functionality (Miller et al. 2011). The structure of the LNS3-EGF2-LNS4 unit in α neurexin is particularly surprising because it reveals a reelin-like domain arrangement (Chen et al. 2011), a protein domain involved in neuron migration, which may suggest a role for neurexins in this process. In addition, all known calcium binding sites of LNS2 to LNS5 face to one side of the molecule, resulting in a putative regulation of the calcium coordination site of LNS4 by intermolecular contacts to LNS3 (Reissner and Missler 2011). Both groups present a neurexin 1α/neuroligin1 complex model in which the neuroligin splice insert A comes in steric contact with the LNS4 domain and with the α-neurexin splice insert at #SS3, if it is present. Moreover, α-neurexins promoting GABAergic postsynaptic specialization activity is mediated by the LNS6 domain and negatively modulated by upstream sequences in the LNS3, LNS4, and the intervening EGF-like domain out lighting (Kang et al. 2008). We have recently found that downregulation of Nrxn2α E11-included transcripts decrease the inhibitory synapse scaffold protein Gephyrin in the circadian clock (Shapiro-Reznik et al. 2012) linking this exon balance of excitatory/inhibitory neurotransmission. Altogether, these studies suggest that an insert at #SS3 has an important role in neurexins functional activity and subsequently synaptic specification.

Therefore, understanding the regulation mechanism of exon 11 NRXN2α splicing may help to define the molecular control of dynamic, activity-dependent changes in synaptic strength that occur in the adult brain.

The data presented here demonstrate that hnRNP L is essential for membrane depolarization to regulate the alternative splicing of NRXN2α exon 11. Like cortical neurons, KCl-induced membrane depolarization of Neuro2A cells resulted in NRXN2α E11 splicing repression. Neuro2a cells are therefore a useful model to explore *cis* elements and *trans*-acting factors regulating the depolarization-induced NRXN2α pre-mRNA E11 alternative splicing. Four independent and complementing approaches were used in this analysis: in silico analysis of putative exonic *cis* elements, mutagenesis to identify *cis* elements residing in E11, E11-RNA pull-down to identify *trans*-acting factors, and siRNA mediated downregulation to confirm the role of the identified factors.

Typically, silencers and enhancers are present within the vicinity of potential exon/intron junctions, suggesting that the interplay between activating and repressing *cis*-acting elements modulates the probability of exon inclusion (Black 2003). Accordingly, whereas the 5′ region of the exon appears to be implicated in the repression of E11 splicing, the 3′ region seems to have an active role in the inclusion of E11 in NRXN2α transcripts. In particular, expression of E11 included transcripts was markedly reduced by the M2 and M3 mutations targeting the CTGCCTG and CGCGTC elements, respectively, that area putative SR proteins family (SF2/ASF, SRp40, SRp55) binding motif. The M4 mutation that targets GCACCCA motif located in close proximity to the 3′-splice site enhanced E11 splicing leading to preference of exon 11 inclusion under basal conditions. The GCACCCA motif has a single short C-stretch which is sufficient for maximal high affinity hnRNP K binding (Thisted et al. 2001) and a CACC sequence previously identified as an hnRNP L target motif (Hui et al. 2005). Moreover, the NRXN2α E11 CACC motif is shared by NRXN2α E11, NRXN2α E2, and NRXN2α E3, all of which demonstrate depolarization-induced splicing repression (Rozic-Kotliroff and Zisapel 2007).

Whereas M4 mutation still allowed for the depolarization-induced splicing repression to occur, M2 mutation targeting the CTGCCTG element obviated the depolarization-induced splicing repression. These results thus indicate putative exon 11 motifs that are involved in depolarization-induced E11 NRXN2 splicing regulation.

The pull-down analysis identified the hnRNP K and hnRNP L binding to E11 and indicated that the binding of hnRNP L to E11 was enhanced in depolarized cells. Importantly, depolarization does not affect the levels of the hnRNP L and hnRNP K transcripts in the Neuro2a cells. The critical role of hnRNP K and hnRNP L in E11 splicing regulation was confirmed by knockdown experiments in Neuro2a cells, demonstrating that reduction in hnRNP L and hnRNP K enhanced E11 splicing under basal conditions thus increasing the amount of NRXN2α E11 including transcripts.

Our bioinformatics and E11 mRNA binding experiments show that an SR protein (SRp40) is an E11 mRNA binding protein. As with hnRNP K and hnRNP L, there were no changes in the level of SRp40 mRNA transcripts under low and high KCL conditions (data not shown). The level of E11 mRNA-bound SRp40 decreased upon depolarization but so were levels of a number of other proteins. Because SRp40 is a putative splicing enhancer, the decrease in E11 binding in depolarized cells is compatible with an opposite function to hnRNP K/L. The exact role of SRp40 in E11 splicing remains to be investigated. Accumulating evidence supports a role of hnRNP K (or family members) and hnRNP L in activity-dependent alternative splicing regulation. The first RNA element shown to confer an exon-skipping response to KCl-mediated depolarization was found on the STREX exon of the BK potassium channel. This element was identified as CaMKIV-responsive RNA elements (CaRRE1 and CaRRE2) or the UAGG motif (Lee et al. 2007). Recently, it has been shown that the regulation of the STREX exon by membrane depolarization requires a highly conserved CaMKIV target serine (Ser513) whose phosphorylation enhanced hnRNP L interaction with the CaMKIV RNA element 1 of STREX(Liu et al. 2012). In addition, hnRNP L has been identified as an essential factor mediating the membrane depolarization/CaMKIV-regulated alternative splicing of this exon through short CA repeat elements at or in close proximity to the 3′-splice site (Yu et al. 2009). More recently, a member of the hnRNP K family (SAM68) has been demonstrated to regulate activity-dependent alternative splicing at exon 20 of NRXN1 gene (Iijima et al. 2011). The identified RNA binding proteins may participate also in controlling activity-dependent alternative splicing of other neuronal pre-mRNAs (Sharma and Lou 2011).

Notably, despite the fact that depolarization of the Neuro2a cells increased the amounts of E11 bound hnRNP L without affecting the binding of hnRNP K, the knockdown of either hnRNP K, like that of hnRNP L, increased the amount of E11 included transcripts and obviated the depolarization-induced E11 splicing repression. As shown here, NRXN2α E11 splicing is mediated via two distinct exonal motifs that target multiple enhancers and repressors. It is thus possible that hnRNP K and hnRNP L act in concert with enhancers in a combinatorial mode to regulate signal-induced E11 splicing. This conclusion is compatible with the role of these proteins in alternative splicing in other genes. For example, ESS1 splicing silencer in CD45 exon 4 confers basal exon skipping in resting T cells via hnRNP L and activation-induced exon skipping in activated T cells via PSF. Activation-induced posttranslational modification of hnRNP L correlates with a modest increase in the protein’s repressive activity and the splicing factor PSF is recruited to the ESS1 complex in an activation-dependent manner and accounts for the majority of the signal-regulated ESS1 activity (Melton et al. 2007). Such combinatorial effects on splicing allows for precise regulation of signal-induced alternative splicing.

It has already been shown that the posttranslational modifications of hnRNP K and hnRNP L affect their cellular activities conferring to them the dual ability to promote both exon skipping and exon inclusion events (Venables et al. 2008). Recent studies indicated that multiple alternative forms of hnRNP K produced mainly by alternative splicing of the single hnRNP K gene and by phosphorylation are implicated in cell regulation and signal transduction (Kimura et al. 2010). The possibility that combinatorial regulation is mediated via hnRNP K and/or hnRNP L phosphorylation warrants further investigations.

The hnRNP K protein is involved in various cellular processes, such as chromatin remodeling, transcription, mRNA processing, and translation and interacts with a wide range of binding partners through multiple domains. In this study, beside regulation of E11 splicing, hnRNP K depletion enhanced innate NRXN2α expression in the cells suggesting that under basal condition hnRNP K effects translational repression. There are several examples that phosphorylated hnRNP K acts as a translational repressor for other genes (Habelhah et al. 2001a, b; Ostareck-Lederer et al. 2002). In this context, phosphorylation of hnRNP K (whether via ROCK or PKC activation) might explain the decrease in NRXN2α expression in depolarized Neuro2A cells. However, such translational repression might be mediated via interaction at sites other than the exons 10, 11, and 12 because it is not seen with the minigene mRNA.

In summary, the present study has established by mutational analysis and RNA binding assays a key role of exonal motifs CTGCCTG (enhancer) and CACC (suppressor) in regulating E11 NRXN2α basal and activity-dependent alternative splicing. It also identified hnRNP L and hnRNP K as suppressors of NRXN2α E11 alternative splicing with a key role in depolarization-induced splicing regulation. Because the spliced exon belongs to the extracellular domain of NRXN2α, the enhancement and suppression of its splicing may have important implications with respect to *trans*-synaptic signaling of the protein.
